# Effects of monitoring exercise rehabilitation with target intensity on the patient with twice PCI: A case report

**DOI:** 10.1097/MD.0000000000033583

**Published:** 2023-04-21

**Authors:** Xiangyang Liu, Yunxian Chen, Jinfeng Chen, Aihua Li, Ming Zhong, Wanming Zhou, Liangqiu Tang

**Affiliations:** a Department of Cardiology, Yuebei People’s Hospital Affiliated To Shantou University, Shaoguan, Guangdong, China.

**Keywords:** cardiopulmonary exercise testing, case report, exercise rehabilitation, flow-mediated dilation, lipoprotein-associated phospholipase A2, percutaneous coronary intervention

## Abstract

As the core of cardiac rehabilitation (CR), early exercise rehabilitation is beneficial for patients with coronary heart disease (CHD), and center-based CR with target intensity is superior to home-based CR. However, there was no research to observe the effects of exercise rehabilitation on cardiopulmonary exercise capacity, oxygen uptake efficiency slope, endothelial function evaluated as flow-mediated vasodilation (FMD), and blood plasma lipoprotein-associated phospholipase A2 (Lp-PLA2) in CHD patients undergone percutaneous coronary intervention (PCI) for 3 months.

**Patient concerns:**

A 57-year-old woman had been identified with triple vessel disease and undergone twice PCI for complete revascularization, however, there was no improvement in Lp-PLA2, FMD, and related indicators of cardiopulmonary exercise testing.

**Diagnosis:**

Coronary angiography showed an 85% stenosis in the middle left anterior descending artery, an 85% stenosis in the proximity of a thick first-diagonal branch, a long 75 to 85% stenosis in the middle left circumflex artery, and a 90 to 95% stenosis in the proximal. The case was diagnosed as CHD.

**Interventions:**

The patient obtained optimal medical therapy comprising therapeutic lifestyle changes, and began monitoring exercise rehabilitation with target intensity 3 months after the second PCI in the CR center.

**Outcomes:**

There were changes in cardiopulmonary exercise capacity, oxygen uptake efficiency slope, FMD, and Lp-PLA2 in the patient with 3 apparent stenotic coronary arteries who was done PCI twice, without or with postoperative exercise rehabilitation, respectively.

**Lessons:**

We proved that monitoring exercise rehabilitation training with target intensity could improve the prognosis of chronic coronary syndrome patients, and it was never too late to do regular exercise rehabilitation.

## 1. Introduction

At present, optimal medical therapy (OMT)^[1]^ and timely coronary revascularization^[2]^ continue to improve the prognosis of coronary heart disease (CHD) patients. However, angina pectoris, myocardial infarction, malignant arrhythmia, heart failure, and mortality remain high due to coronary artery stenosis or occlusion. Cardiac rehabilitation (CR) in CHD patients arises at a historic moment with the implementation of comprehensive management, and exercise rehabilitation is the core of CR.^[3]^ The meta-analysis had shown that exercise-based CR could improve angina pectoris, myocardial infarction, and restenosis in people with CHD.^[4,5]^ Compared with other forms of exercise rehabilitation, only center-based CR significantly reduced all-cause mortality.^[6]^ In all the studies on CHD with exercise rehabilitation, the patients started exercise rehabilitation within 1 month after receiving OMT or OMT combined with coronary revascularization. There were few research or case reports on patients’ center-based exercise rehabilitation 3 months after coronary revascularization.

## 2. Case description

History of present illness: A 57-year-old woman presented to the Department of Cardiology with worsening chest tightness and dyspnea during the activity. She had chest tightness and dyspnea located in the middle of her chest 2 years ago. The chest tightness showed paroxysmal squeezing and could be relieved after the break. The patient did not treat systematically. A month ago, the patient felt the chest tightness and dyspnea were worse than before during the activity. She has been suffering from hypertension for 4 years, and her blood pressure was up to 200/110 mm Hg when untreated. She has been taking 40 mg captopril once a day and 12.5 mg Metoprolol Tartrate Tablets twice daily to maintain her blood pressure under 140/90 mm Hg daily.

Examination: On physical examination at admission, the patient’s temperature was 36.3°C, pulse and beat rhythm 62 bpm, respiration rate 20/min, and blood pressure 118/75 mm Hg. She was conscious and lucid. The lungs displayed clear breathing sounds without dry or wet rale or pleural rub. The beat rhythm was tidy, and there was no noise in each valve auscultation area. Her abdomen was soft without the pain of the pressure or rebound or muscle tension.

**Figure 1.**
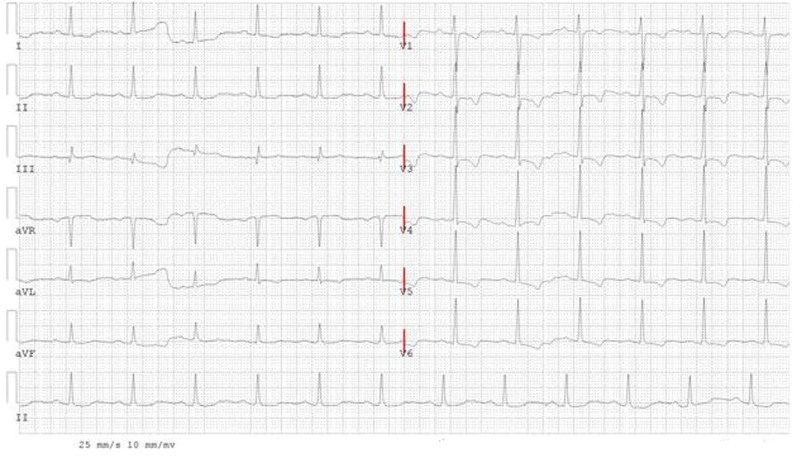
Electrocardiogram of the first admission.

**Figure 2.**
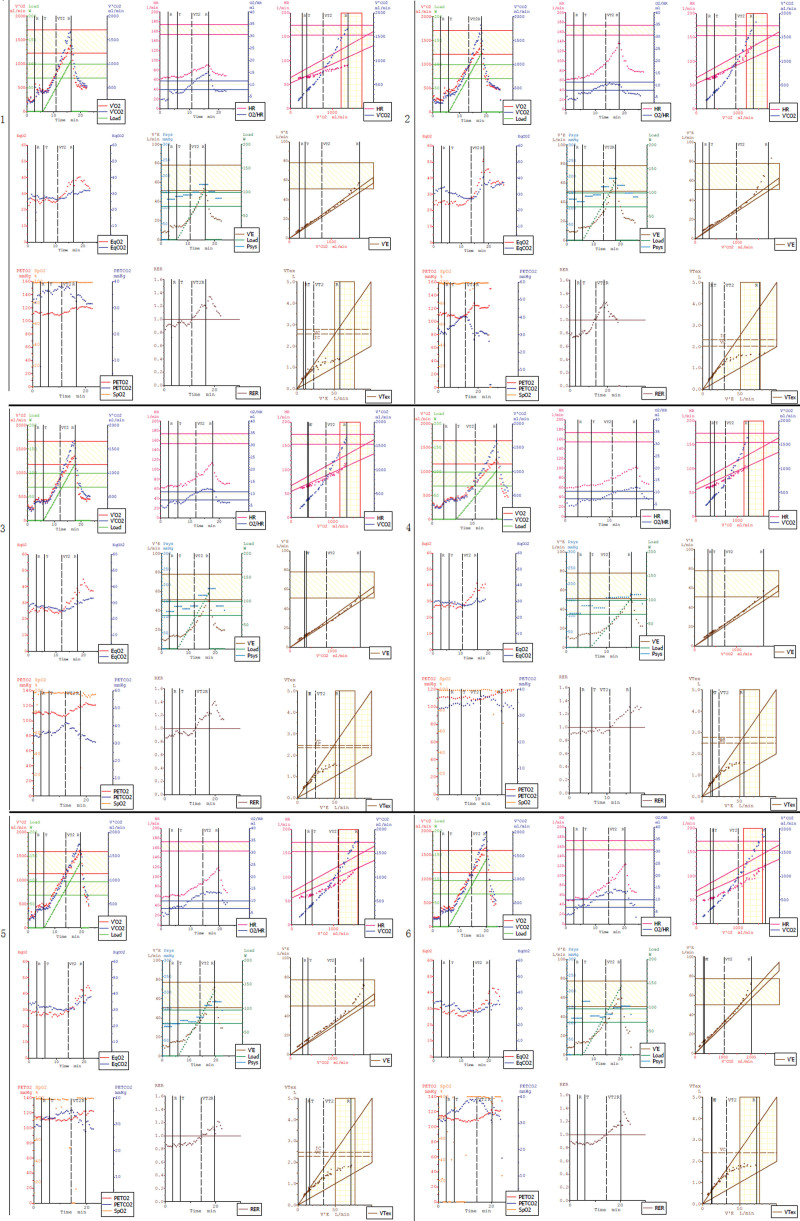
The new 9-panel plot of cardiopulmonary exercise testing.

Investigation: Blood routine, myocardial enzyme index, troponin, and plasma D2 polymer were regular. Blood lipids were total cholesterol 4.07 mmol/L, 3 acyl glycerin 3.94 mmol/l, high-density lipoprotein cholesterol 1.00 mmol/L, and low-density lipoprotein cholesterol 2.44 mmol/L. The level of lipoprotein-associated phospholipase A2 (Lp-PLA2) with 298 ng/mL was high. The flow-mediated vasodilation (FMD) was 1.4% (Table 1). The electrocardiogram (ECG) demonstrated sinus rhythm, the first-degree atrioventricular block, ST-segment depression, and T wave inversion (Fig. 1). The first cardiopulmonary exercise testing (CPET) showed that exercise capacity and small airway function were abnormal. The patient had not finished the ECG exercise test, and there was no arrhythmia. The partial parameters of CPET showed in (Table 1, Fig. 2). Coronary angiography (CAG) showed no stenosis with the left main trunk, an 85% stenosis in the middle left anterior descending artery (LAD), an 85% stenosis in the proximity of thick first-diagonal branch (D1), a long 75 to 85% stenosis in the middle left circumflex artery (LCX), and a 90 to 95% stenosis in the proximal and middle right coronary artery (RCA) (Fig. 3.1a and 1b).

**Table 1:** Comparison of clinical parameters and follow-up data for the patient.

	November 25, 2020	December 30, 2020	March 5, 2021	May 26, 2021	September 6, 2021	April 15, 2022
TC (mmol/l)	4.07	3.22	3.57	3.24	2.95	3.02
TG (mmol/l)	3.94	0.78	0.48	0.67	0.51	0.58
HDL-C (mmol/l)	1	1.03	1.37	1.13	1.23	1.32
LDL-C (mmol/l)	2.44	1.83	1.89	1.73	1.2	1.43
Lp-PLA2 (ng/mL)	298	–	350	481	206	100
FMD	1.4	6.9	3.1	6.4	6.9	8.3
RER	1.2	1.22	1.2	1.29	1.19	1.21
Peak VO_2_ (mL/min/kg)	18.5	20.4	20.4	19.4	25.7	26.9
VO_2_ at AT (mL/min/kg)	10.3	12.6	12.3	10.5	17.3	17.8
Mets	5.3	5.8	5.8	5.6	7.4	7.7
OUES (mL/min log/L/min)	1312	1352.27	1489.23	1342.4	1606.84	1637.14

FMD = flow-mediated vasodilation, HDL-C = high-density lipoprotein cholesterol, LDL-C = low-density lipoprotein cholesterol, LP-PLA2 = lipoprotein-associated phospholipase A2, Mets = metabolic equivalents, OUES = oxygen uptake efficiency slope, Peak VO2 = peak oxygen consumption, RER = respiratory exchange ratio, TC = total cholesterol, TG = 3 acyl glycerin, VO2 at AT = oxygen consumption at anaerobic threshold.

The electrocardiogram demonstrated sinus rhythm, the first-degree atrioventricular block, ST-segment depression, and T-wave inversion.

(2.1, 2.4, 2.5, and 2.6) The O2/HR was normal in the middle of the exercise of the first, fourth, fifth, and sixth CPET. (2.2) The O2/HR fell in the middle of the exercise of the second CPET and (2.3) flatted in the middle of the exercise of the third CPET. CPET = cardiopulmonary exercise testing.

**Figure 3.**
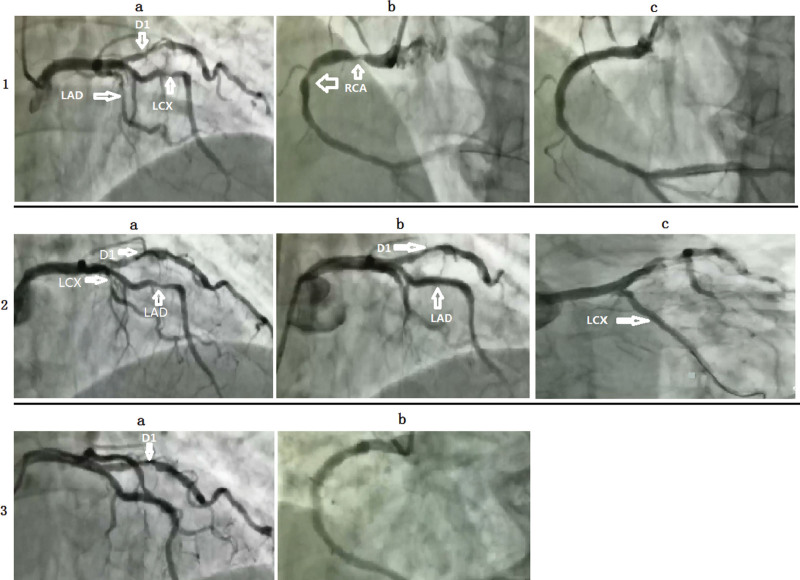
Coronary angiography of the patient.

(1a) An 85% stenosis in the middle LAD (white arrow), an 85% stenosis in the proximity of thick D1 (white arrow), and a long 75 to 85% stenosis in the middle LCX (white arrow). (1b) A 90 to 95% stenosis in the proximal and middle RCA (white arrow). (1c) A DES was implanted in stenosis RCA. (2a) An 85% stenosis in the middle LAD (white arrow), an 85% stenosis in the proximity of thick D1 (white arrow), and a long 75 to 85% stenosis in the middle LCX (white arrow). (2b) An 85% stenosis in the proximity of thick D1 (white arrow) and a DES was implanted in stenosis of LAD. (2c) A DES was implanted in stenosis of LCX. (3a) A 70% stenosis in the proximity of thick D1. (3b) No stenosis in the RCA. D1 = first-diagonal branch, DES = drug-eluting stent, LAD = left anterior descending artery, LCX = left circumflex artery, RCA = right coronary artery.

Diagnosis: Chronic coronary syndrome (CCS).

**Figure 4.**
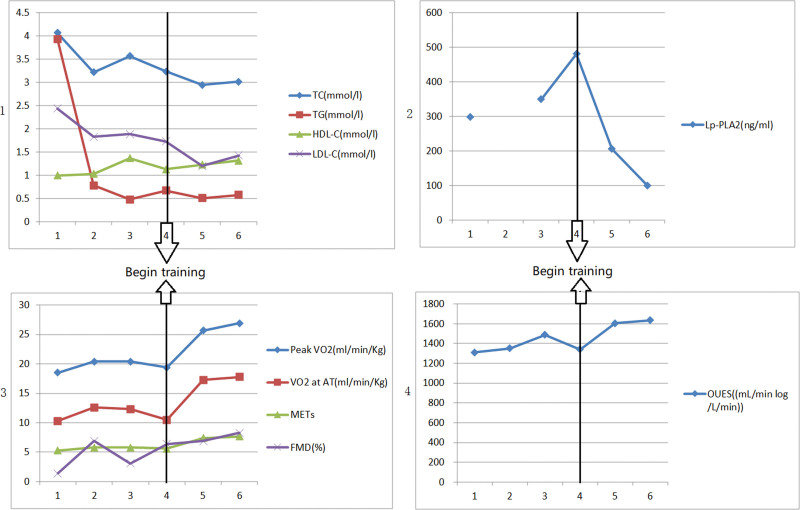
The line chart of the patient’s all indicators.

Treatment: (OMT/ percutaneous coronary intervention (PCI)/exercise rehabilitation training). After admission, the patient obtained OMT comprising of therapeutic lifestyle changes as follows: Aspirin 100 mg once a day, clopidogrel 75 mg once a day, Rosuvastatin 10 mg at bedtime, metoprolol 6.25 mg 2 times per day, telmisartan 40 mg once a day, and nicorandil 5 mg 3 times a day. According to the CAG, we implanted a drug-eluting stent (DES) (3.5 mm × 30 mm, Firebird2) in the proximal and middle RCA (Fig. 3.1c). After 1 month, we reevaluated FMD (Table 1) and CPET (Table 1, Fig. 2.2). Blood lipid (Table 1), Lp-PLA2 (Table 1), FMD (Table 1), and CPET (Table 1, Fig. 2.3) were reevaluated after 3 months. The patient’s O2/HR fell in the middle of the exercise of the second CPET (Fig. 2.2) and flatted in the middle of the exercise of the third CPET (Fig. 2.3), which showed that the patient remained myocardial ischemia^[7]^ because of severe coronary artery stenosis. CAG reevaluated showed no stenosis with the left main trunk and the stent of RCA, an 85% stenosis in the middle LAD, an 85% stenosis in the proximal D1, a long 75 to 85% stenosis in the middle LCX (Fig. 3.2a). Then we implanted a DES (2.75 mm × 23 mm, Firebird2) in the lesion of LAD and a DES (2.5 mm × 29 mm, Firebird2) in the lesion of LCX (Fig. 3.2b and 2c). We continued to advise patients to keep low salt, low-fat diet, and exercise training without an exercise prescription. The same parameters were reevaluated about 3 months after the second PCI (Table 1, Fig. 2.4). There was no improvement in Lp-PLA2, FMD, and related indicators of CPET. According to the result of CPET, combined with the power and heart rate on the anaerobic threshold, we made the exercise prescription for the patient. She began monitoring exercise rehabilitation with target intensity in the CR center 3 times per week. Combined with home-based exercise training, the total number of patients’ exercise rehabilitation arrived 5 to 7 times per week. The same parameters were reevaluated 6 months after the second PCI (Table 1, Fig. 2.5). There was an obvious improvement in Lp-PLA2, FMD, and related indicators of CPET. We adjusted the exercise prescription according to CPET. She continued monitoring exercise rehabilitation training with target intensity in the CR center once per week combined with home-based exercise training 4 to 6 times per week. The aim of once-per-week exercise rehabilitation in the CR center was to assess the effect of home-based exercise training so that we could dynamically adjust the exercise prescription. We evaluated the same parameters about 1 year after the second PCI (Table 1, Fig. 2.6), which showed further improvement. There was no stenosis in the RCA, LCX, and LAD Fig. 3.3a and 3b. At the same time, a 70% stenosis in the D1 was better than before (Fig. 3.3a). We provided the patient’s line chart of all indicators (Fig. 4) and a detailed timeline of therapy (Table 2).

**Table 2:** The patient’s detailed timeline of therapy.

Timeline	Events	Intervention
Nov. 2020	Typical angina symptoms	Drug therapy and selective PCI of RCA
Dec. 2020	About 1-month follow-up: no symptom	Drug therapy
Mar. 2021	About 3-month follow-up: no symptom	Drug therapy, selective PCI of LAD and LCX
May 2021	About 6-month follow-up: no symptom	Drug therapy and exercise rehabilitation
Sept. 2021	About 9-month follow-up: no symptom	Drug therapy and exercise rehabilitation
Apr. 2022	About 15-month follow-up: no symptom	Drug therapy and exercise rehabilitation

LAD = left anterior descending artery, LCX = left circumflex artery, PCI = percutaneous coronary intervention, RCA = right coronary artery.

The horizontal axis 1 = the first PCI preoperatively, 2 = 1 month after the first PCI, 3 = 3 months after the first PCI and the second PCI preoperatively, 4 = 3 months after the second PCI, 5 = 6 months after the second PCI, 6 = 1 year after the second PCI. FMD = flow-mediated vasodilation, HDL-C = high-density lipoprotein cholesterol, LDL-C = low-density lipoprotein cholesterol, LP-PLA2 = lipoprotein-associated phospholipase A2, Mets = metabolic equivalents, OUES = oxygen uptake efficiency slope, Peak VO2 = peak oxygen consumption, PCI = percutaneous coronary intervention, TC = total cholesterol, TG = three acyl glycerin, VO2 at AT = oxygen consumption at anaerobic threshold.

## 3. Discussion

Elective revascularisation was recommended for CCS patients with severe stenosis arteries,^[8]^ and patients who had undergone PCI were better than seemingly similar patients who had not.^[9]^ The COURAGE trial and ISCHEMIA trial prompted that PCI was effective at reducing ischemia. Nevertheless, it did not affect hospitalization caused by heart failure, unstable angina, myocardial infarction, and death from cardiovascular causes.^[10,11]^ As the core of CR, exercise rehabilitation could improve cardiopulmonary exercise capacity,^[12]^ oxygen uptake efficiency slope,^[13]^ FMD^[14]^ and reduce inflammation,^[15]^ the incidence of coronary restenosis,^[16]^ overall mortality and cardiac mortality in CHD patients undergoing PCI.^[5]^ Because of the more active chest tightness than before and myocardial ischemia showed by ECG, combined with the ischemia area showed by ECG and the CAG, we implanted a DES in the patient’s proximal and middle RCA. In addition, we advised her to undergo selective PCI if she still had chest discomfort combined with the CAG. She obtained OMT after PCI. We required her to low salt, low-fat diet. Furthermore, we advised her to do center-based CR with target intensity which was superior to home-based CR. However, the patient rejected exercise training in the CR center. Because there was no significant improvement in cardiopulmonary exercise capacity and fall or flattening in O2/HR,^[7]^ which suggested the myocardial ischemia in the middle of the exercise of the CPET reevaluated at the first and third month after PCI, we separately implanted a DES in the lesion of LAD and LCX after further communication with the patient and family members. However, the indicators of CPET reevaluated 3 months after the second PCI showed a decline, similar to the index before the first PCI. That was to say, OMT and twice PCI did not improve her cardiopulmonary exercise capacity and reduce the level of Lp-PLA2. All of these indicated that the prognosis of the patients might not be improved. The FMD had improved in the first month after the first PCI; however, it did not continue to improve in the third month after the second PCI. This change mainly might be attributed to taking statins.^[17]^ The patient began regularly monitoring exercise rehabilitation guided by exercise prescription, and there was a noticeable improvement in cardiopulmonary exercise capacity, oxygen uptake efficiency slope, metabolic equivalents at peak, FMD, and Lp-PLA2. All of these indicated that the prognosis of the patients might be improved.^[11–13,18,19]^ According to the third CAG, the stenosis of D1 and blood flow were better than before, which suggested that coronary stenosis and blood flow improved by exercise rehabilitation.^[20,21]^ Besides, O2/HR fell at the end of the last CPET, which revealed that we should regularly assess the cardiopulmonary exercise capacity by CPET and adjust exercise prescription. In addition, we should advise patients to do monitoring exercise rehabilitation so that we could reduce the risk and improve the effectiveness of exercise rehabilitation, even for CHD patients whose cardiopulmonary exercise capacity was improved significantly.

## 4. Conclusion

In this case, by comparing the indicators of the patient who have undertaken PCI before and after exercise rehabilitation, we proved that to improve the prognosis of CCS patients, they should obtain the therapy of OMT and PCI and also do monitoring exercise rehabilitation training with target intensity. It was never too late to do regular exercise rehabilitation.

## Acknowledgments

The authors are grateful to Huan Ma (Doctor of Medicine & Director, Department of Cardiac Rehabilitation, Guangdong Cardiovascular Institute, Guangdong Provincial People’s Hospital, Guangdong Academy of Medical Sciences, Guangzhou) and Huaibin Wan (Doctor of Medicine & Chief Physician, Dean of Heyuan Research Center for Cardiovascular Diseases, Department of Cardiology, The Fifth Affiliated Hospital of Jinan University) for giving suggestions on the draft of the paper, and Songhui Xu (Lecturer, School of Foreign Languages, Yanshan University) for helping with polishing and checking the English translation. Author details: Department of Cardiology, Yuebei People’s Hospital Affiliated to Shantou University, Shaoguan 512026, Guangdong, China.

## Author contributions

**Conceptualization:** Xiangyang Liu, Liangqiu Tang.

**Data curation:** Xiangyang Liu, Yunxian Chen, Jinfeng Chen.

**Investigation:** Xiangyang Liu, Ming Zhong, Wanming Zhou.

**Resources:** Yunxian Chen, Jinfeng Chen.

**Writing – original draft:** Xiangyang Liu.

**Writing – review & editing:** Aihua Li, Liangqiu Tang.
